# Developing Online Recruitment and Retention Methods for HIV Prevention Research Among Adolescent Males Who Are Interested in Sex with Males: Interviews with Adolescent Males

**DOI:** 10.2196/jmir.8959

**Published:** 2017-12-21

**Authors:** Kimberly M Nelson, Jaime J Ramirez, Michael P Carey

**Affiliations:** ^1^ Centers for Behavioral and Preventative Medicine The Miriam Hospital Providence, RI United States; ^2^ Department of Psychiatry and Human Behavior Brown University Providence, RI United States; ^3^ Department of Behavioral and Social Sciences Brown University Providence, RI United States

**Keywords:** HIV, adolescent males, sexual minority, recruitment, retention, online research, MSM

## Abstract

**Background:**

Adolescent males interested in sex with males (AMSM) are an important audience for HIV prevention interventions, but they are difficult to reach due to their age and social stigma.

**Objective:**

We aim to identify efficient methods to recruit and retain AMSM in online research.

**Methods:**

Interviews with 14-to-18-year-old AMSM (N=16) were conducted at 2017 Pride events in Boston, MA and Providence, RI.

**Results:**

Participants reported that (1) social media platforms are viable recruitment venues; (2) recruitment advertisements should describe the study using colorful/bright pictures, familiar words, and information about compensation; (3) surveys should be <20 minutes in length; (4) modest compensation (eg, email gift card, US $10 to $20) was preferred; and (5) communications that remind participants about the length and content of surveys, and compensation, should be sent between study activities to increase retention.

**Conclusions:**

Soliciting input from AMSM provides critical guidance regarding recruitment and retention procedures to increase the efficiency of HIV prevention research for this at-risk group.

## Introduction

Adolescent males who are interested in sex with males (AMSM) are at increased risk for HIV in the United States [1]. Elevated HIV rates among AMSM are attributed to early sexual experiences and behavior patterns formed during adolescence [2,3]. Despite evidence of considerable sexual risk-taking [2] and HIV risk among AMSM [1], there is a paucity of HIV research and interventions targeting AMSM [4]. HIV prevention interventions targeting AMSM during adolescence (before or shortly after their sexual debut) can help them establish healthy sexual behaviors, which will have both short- and long-term benefits [3,5,6].

Social networking websites/phone apps are increasingly used to recruit and retain difficult-to-reach populations in health research [7]. AMSM use the Internet and mobile technology to explore their sexuality and seek resources, so social networking websites/phone apps are particularly important for reaching and providing interventions to AMSM [4]. Community engagement is an important part of the development of social network-based recruitment/retention strategies for online sexual health and HIV prevention interventions [8], and has been successfully used with adult men who have sex with men (MSM) and youth [9,10]. Although two online HIV intervention studies have successfully recruited and retained samples that include MSM <18 years old [11,12], only one study focused exclusively on AMSM (aged 14-18 years) [13]. We were unable to find any studies that inquired with AMSM themselves about optimal online recruitment and retention methods. The current study sought to fill this gap; specifically, interviews were conducted with 14-to-18-year-old AMSM to understand acceptable ways to recruit and retain them in online HIV prevention research. By having AMSM inform these techniques, researchers will be better prepared to successfully conduct online HIV prevention research and develop online interventions to address HIV disparities among AMSM.

### Methods

A convenience sample of AMSM (N=16) were interviewed during 2017 Boston and Rhode Island Pride events. Potential participants were approached by study staff who briefly described the study and established eligibility. Eligibility criteria included: (1) being 14-to-18 years old; (2) being cis-male; and (3) identifying as gay/bisexual, reporting sexual attraction to male partners, or reporting voluntary sexual contact with a male partner in the past year. Youth provided verbal assent or consent, depending on their age, and completed a capacity to consent assessment. A waiver of guardian permission was obtained.

Forty-four individuals agreed to be screened; of those, 16 (16/44, 36%) were eligible, and of those all (16/16, 100%) agreed to participate. Of the 28 individuals who were ineligible, the most common reasons for ineligibility were not identifying as cis-male (15/28, 54%) and being over 18 years of age (12/28, 43%). Interviews took approximately 20 minutes to complete. Answers were recorded on smartphones using REDCap [14] and data were coded in real-time by the interviewers. The interview was programed with a list of potential answers for each question, as well as an open-ended response option to capture responses that fell outside the listed options. For open-ended responses, a framework matrix analysis was conducted after data collection was complete [15,16].

Participants were informed as a part of the assent/consent process that the questions they were being asked were part of a larger online sexual health study. Specifically, they were told, “the larger study is designed to determine if an online-delivered sexual health education program might help young men like you stay healthy and avoid sexually transmitted diseases.” For recruitment, participants were asked which social media websites/phone apps they used, the one they used most frequently, and which ones would be good to advertise on. Respondents were asked about the features that would be important to include in online advertisements and, of those features, which would be the most important to include. For retention, participants were asked about the longest online survey they would be willing to complete, ways to increase retention in a longitudinal online study, preferred forms of compensation, and their preferred compensation amount for a 30-minute online survey. All procedures were approved by The Miriam Hospital Institutional Review Board. Participants received US $5 for the interview.

## Results

The average age of the sample was 16 years (standard deviation=2). Eight participants (8/16, 50%) identified as a racial/ethnic minority, 11 participants (11/16, 69%) were gay-identified, and 12 participants (12/16, 75%) reported being sexually active with a male partner in the last year.

For recruitment (Table 1), the majority of respondents used Facebook (15/16, 94%), YouTube (14/16, 88%), Instagram (13/16, 81%), and Snapchat (13/16, 81%). The most frequently used websites/phone apps were Facebook (5/16, 31%) and Snapchat (5/16, 31%). The majority of participants said Instagram (12/16, 75%), Snapchat (11/16, 69%), and Facebook (10/16, 63%) would be the best websites/phone apps to advertise on. Participants reported that it was important for online advertisements to include a brief description of the study with short phrases and bullet points (10/16, 63%), colorful and bright pictures (9/16, 56%), information about compensation (9/16, 56%), and familiar/comfortable words (8/16, 50%). Among these options, colorful and bright pictures (4/16, 25%) and a short description (3/16, 19%) were identified as most important.

For retention (Table 2), the majority of respondents (9/16, 56%) would complete a 10-to-20-minute survey. Information about compensation (11/16, 69%), the length of surveys (10/16, 63%), and what kinds of questions would be asked (10/16, 63%) were identified as important information to provide. The majority of respondents said that sending reminders between study activities (15/16, 94%) and providing compensation (12/16, 75%) would increase retention. Participants preferred an email gift card (11/16, 69%) as compensation. More than half of the participants (9/16, 56%) said that US $10-$20 is fair compensation for a 30-minute online survey.

**Table 1:** Website use and online recruitment strategies identified by AMSM (N=16).

Website use and recruitment strategies		Total, n (%)
**Websites used by AMSM**		
	Facebook	15 (94)
	YouTube	14 (88)
	Instagram	13 (81)
	Snapchat	13 (81)
	Twitter	4 (25)
	Tumblr	7 (44)
	Pinterest	3 (19)
	Google+	1 (6)
**Best websites for advertising/recruiting AMSM**		
	Instagram	12 (75)
	Snapchat	11 (69)
	Facebook	10 (63)
	Twitter	6 (38)
	YouTube	5 (31)
	Tumblr	5 (31)
	Pinterest	1 (6)
	Vine	1 (6)
**Style and content features for online advertisements**		
	A brief description with short phrases and bullet points	10 (63)
	Colorful and bright pictures	9 (56)
	How they will be compensated	9 (56)
	Familiar and comfortable words	8 (50)
	If parent/guardian permission is required or not	6 (38)
	A detailed description of the study	4 (25)
	How long the study will take	3 (19)

**Table 2:** Retention strategies identified by AMSM (N=16).

Retention strategies		Total, n (%)
**Longest online survey willing to fill out**		
	5-10 minutes	6 (38)
	10-20 minutes	9 (56)
	>20 minutes	1 (6)
**Information to include in study materials to increase retention**		
	How participants will be compensated	11 (69)
	Length of the survey	10 (63)
	Content (ie, what will be asked)	10 (63)
	Importance of the questions	5 (31)
	Confidentiality assurances	1 (6)
**Ways to increase retention in longitudinal online studies**		
	Send reminders between study activities (eg, phone calls, text messages, or emails)	15 (94)
	Provide compensation	12 (75)
	Increase compensation value in increments for each activity completed	1 (6)
**Preferred forms of compensation**		
	Email gift card	11 (69)
	Check in mail	5 (31)
	Cash in mail	2 (13)
**Preferred compensation amount for a 30-minute online survey (US $)**		
	$10-$20	9 (56)
	$20-$30	7 (44)
	$30+	0 (0)

## Discussion

Facebook advertisements have been the predominant way that online HIV intervention studies have recruited AMSM [11-13]. In addition to Facebook, our results indicate that other social media platforms may also be useful. Specifically, most AMSM in our sample report using multiple social networking websites/phone apps. An assessment of the Facebook advertisements used in the intervention study targeting 14-to-18-year-old AMSM found that having images that are salient to AMSM improved recruitment rates [17]. Similarly, our participants indicated that advertisements including bright and colorful images and words that are familiar to AMSM would be important. Our participants also felt that advertisements should include a brief description of the study with short phrases, bullet points, and information about compensation.

Previous online studies have used varying levels of monetary compensation (US $10-$35 per activity) and emailed gift cards to compensate their participants and increase retention [11-13]. Our results corroborate the value of compensation to increase retention, this range of dollar amounts (depending on the length of the study activity), and the use of emailed gift cards. Although previous studies did not indicate other retention-specific procedures, our participants suggested that sending study reminders between study activities may be an additional way to increase retention. Furthermore, AMSM in our study indicated that informing them about how they will be compensated, the length of the surveys, and what they will be asked could also increase retention. Lastly, participants felt that online assessments should be brief.

A limitation of our study is the small sample size. Nonetheless, these results can inform recruitment and retention procedures in online HIV prevention research targeting AMSM. Additional research testing the suggested recruitment and retention procedures is warranted. Furthermore, as the technological landscape quickly shifts, and because adolescents are early adopters of new technology, it is important to continuously assess the current technologies that youth are using. Ultimately, designing recruitment and retention procedures with the input of the target audience (ie, AMSM) will increase the efficiency, reach, validity, and scientific yield of HIV prevention research. This yield, in turn, can facilitate the development of online HIV prevention interventions for this at-risk group.

## Abbreviations

AMSM: adolescent males who are interested in sex with males
MSM: men who have sex with men
